# Phosphorus requirements of quail chicks for optimizing performance

**DOI:** 10.1016/j.psj.2025.105914

**Published:** 2025-09-29

**Authors:** Mehran Mehri, Masoumeh Bameri, Mahmoud Ghazaghi, Morteza Asghari-Moghadam, Hamid-Reza Behboodi, Mohammad Rokouei

**Affiliations:** a Department of Animal Sciences, Faculty of Agriculture, University of Zabol; Sistan 98613-35856, Iran; b Department of Animal and Poultry Physiology, Faculty of Animal Science, Gorgan University of Agricultural Science and Natural Resources; Gorgan 49138-15739, Iran

**Keywords:** Available phosphorus, Japanese quail, Nonlinear regression, Performance

## Abstract

Phosphorus is a critical nutrient for optimal growth and bone development in Japanese quail chicks. This study was conducted to estimate the dietary requirement for available phosphorus in Japanese quails between 7 and 14 days of age, applying a univariate dose–response model across graded dietary levels (0.15–0.35%). A total of 375 quail chicks were randomly assigned to five dietary treatments with 5 pen replicates and 15 birds each, and performance parameters including body weight gain (G), feed intake (FI), and feed conversion ratio (FCR; feed efficiency) measured. Statistical analysis using broken-line regression models revealed that growth performance and feed efficiency were significantly influenced by dietary AP. The optimal AP requirements estimated from G ranged from 0.288 to 0.316%, while those based on FCR ranged from 0.282 to 0.331%. Considering model fit criteria, the recommended dietary AP requirement for quail chicks between 7 and 14 days of age is approximately 0.28 to 0.33%. These findings provide evidence-based guidance for phosphorus supplementation in early-stage quail nutrition, supporting efficient growth while minimizing excess phosphorus excretion and potential environmental impacts.

## Introduction

Phosphorus is a vital mineral required for optimal growth, skeletal development, and metabolic functions in poultry, including young quail chicks. Alongside calcium, phosphorus constitutes a significant portion of bone tissue and is essential for proper bone mineralization and various physiological processes, such as energy metabolism and acid-base balance (Miller, 1967; Nestler et al., 1948). The importance of phosphorus is particularly pronounced during the early developmental period, specifically from 7 to 14 days of age, when the rapid growth rate of quail chicks intensifies their nutrient demands. Inadequate phosphorus supplementation during this critical window can lead to stunted growth and metabolic disorders, making the determination of precise dietary levels essential (Ghazaghi et al., 2022; Miller, 1967; Sacakli et al., 2005). However, the precise phosphorus requirement for Japanese quail during this specific growth phase remains uncertain. The National Research Council (NRC, 1994) suggested 0.3 % available phosphorus for growing quails, whereas more recent work in meat quail estimated a higher requirement of 0.41 % of the diet (Silva et al., 2009). This discrepancy highlights a knowledge gap and suggests that historical recommendations may not be optimal for modern, fast-growing quail genotypes. Furthermore, factors such as dietary calcium levels and the inclusion of phytase, which improves phosphorus availability, can significantly alter the required amount of inorganic supplementation, adding complexity to feed formulation and underscoring the need for a re-evaluation of this critical nutrient requirement (Alfoteih et al., 2007; Sacakli et al., 2005). To address this uncertainty, dose-response experiments provide a reliable and systematic approach to determining precise dietary requirements. By assessing performance, bone quality, and nutrient utilization across a gradient of phosphorus intake levels, such studies can accurately identify the point at which nutrient inclusion meets physiological demands without resulting in excess waste and environmental concerns (Ahmadi et al., 2025; Ghazaghi et al., 2022). The high rate of endogenous phosphorus losses observed in young quail further reinforces the importance of precisely defining the dietary requirement to prevent deficiencies that could compromise development (Ghazaghi et al., 2022).

Therefore, the need for this study is based on the conflicting existing recommendations and the critical importance of phosphorus during the rapid growth phase of 7- to 14-day-old quail chicks. The objective of the present study was to precisely estimate the dietary phosphorus requirement for quail chicks during this period using a dose-response approach. We hypothesized that increasing dietary phosphorus concentrations would lead to a linear improvement in growth performance and bone mineralization parameters up to a specific requirement level, after which these metrics would plateau. By analyzing this response with broken-line regression models, we seek to provide an updated, evidence-based recommendation for the phosphorus nutrition of young quail chicks.

## Materials and methods

### Ethics statement

The experimental procedures were conducted in accordance with the guidelines of the Iranian Council of Animal Care and were approved by the Research Animal Ethics Committee of the University of Zabol (AEUOZ-2012|U2020-BR).

### Chemical analysis

Feed ingredients were analyzed to determine their chemical composition. Dry matter (DM) was measured according to AOAC (2006, method 930.15), ash (942.05), crude fiber (978.10), ether extract (2003.05), calcium (934.01), phosphorus (965.17), and crude protein (CP; 990.03).

### Birds and experimental diets

A dose-response study was designed to estimate the phosphorus requirements of Japanese quail from 7 to 14 days of age. From hatch to day 6, birds received a standard diet formulated according to NRC (1994) to satisfy all nutritional needs. A basal diet deficient in phosphorus (Table 1) was then prepared, and five experimental diets were formulated with increasing phosphorus levels from 0.15 % to 0.35 % in 0.05 % increments. A total of 375 chicks, weighed on day 7, were randomly assigned to the five dietary treatments, with five pens per treatment and 15 birds per pen in a completely randomized design. Birds had ad libitum access to feed and water, and environmental conditions were maintained at 29 ± 2.45°C, 61 ± 2.25 % relative humidity, with an 18-hour light:6-hour dark photoperiod throughout the trial.

**Table 1:** Composition of the basal diet.

Ingredient	Percent
Corn	49.10
Soybean meal	25.38
Corn gluten meal	11.69
Sand	7.40
Soybean oil	2.15
Limestone	1.88
NaHCO_3_	0.83
L-Lysine HCl	0.45
DL-Methionine	0.27
Vitamin premix	0.25
Mineral premix	0.25
NaCl	0.22
L-Threonine	0.12
Nutrient specifications	
AME (kcal/kg)	2900
CP (%)	24.0
Methionine (%)	0.69
Lysine (%)	1.31
Threonine (%)	0.98
Ca (%)	0.80
Available P (%)	0.15
DEB (mEq/kg)	250

### Performance

Body weight, weight gain, and feed intake were recorded at the pen level from days 7 to 14 to calculate feed conversion ratio. No mortality occurred during the study.

### Statistical analysis

All data were analyzed using the GLM procedure in SAS (2002), with pen averages as the experimental unit in a completely randomized design:

$$y_{ij}=\mu +\tau_{i}+\varepsilon_{ij}$$

where $y_{ij}$ is the response of bird *j* under treatment (dietary phosphorus level) *i*,μ is the overall mean, $\tau_{i}$ is the fixed effect of treatment *i*, and$\;\varepsilon_{ij}$.

Normality was evaluated with the Shapiro–Wilk test (α = 0.05). Linear and quadratic responses to dietary available phosphorus (AP) were evaluated, and AP requirements were estimated using broken-line regression models according to Khosravi et al. (2016):

*Two-slope broken line:*

Linear ascending (or descending)-linear descending (or ascending):

Y=L+U×(R−X)×(X<R)+V×(X−R)×(X>R)

*One-slope broken line:*

Linear ascending (or descending)-plateau:

Y=L+U×(R−X)×(X<R)

Quadratic ascending (or descending)-plateau:

$$Y=L+U\times {\left(R-X\right)}^{2}\times \left(X<R\right)$$

where Y represents the bird's response; L denotes the asymptote for the first segment; U and V correspond to the slopes of the first and second segments, respectively, indicating an increasing or decreasing trend; and R represents the breakpoint, which is considered the AP requirement.

Model selection for each variable prioritized the highest R² and lowest *S*_y_.ₓ (residual standard deviation):

$$S_{y.x}=\sqrt{\frac{SS}{df}}$$

## Results and discussion

**Fig. 1:**
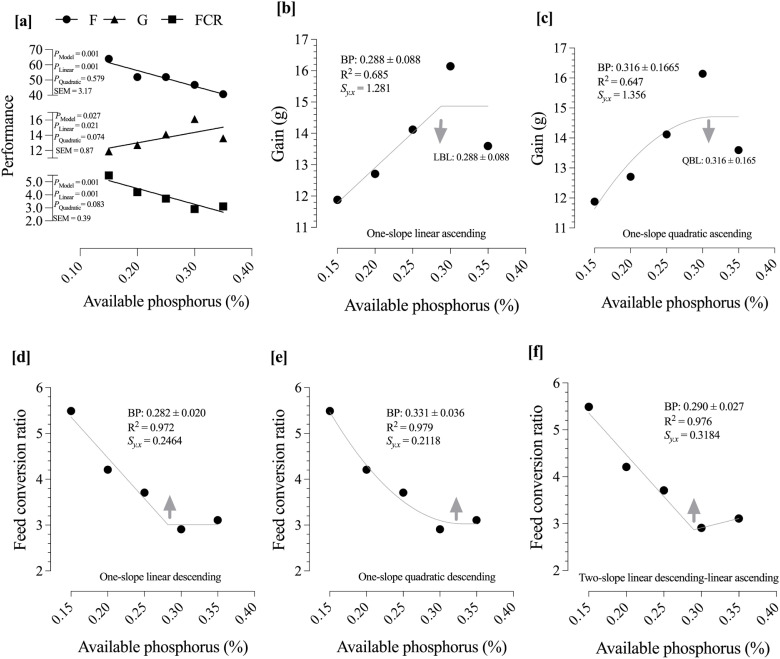
Analysis of variance (a) and broken-line regression analyses to estimate the optimal available phosphorus (AP) level for maximizing weight gain (b, c) and minimizing feed conversion ratio (d–f). Based on the lowest Sy.x and highest R², the best estimates of AP requirement were 0.288 % (95 % CI: 0.1155–0.4605 %) for weight gain and 0.331 % (95 % CI: 0.2604–0.4016 %) for feed conversion ratio.

Growth performance and FCR (or feed efficiency) of growing quails from 7 to 14 days of age were significantly influenced by dietary AP levels (Fig. 1a). Feed intake (*P* = 0.001) responded linearly to increasing dietary AP, while G (linear, *P* = 0.021; quadratic, *P* = 0.074) and FCR (linear, *P* = 0.001; quadratic, *P* = 0.083) showed predominantly linear responses with a tendency toward quadratic effects. Broken-line regression analyses were used to estimate the requirement of available phosphorus (Figs. 1b–F). Based on G, the one-slope linear ascending model estimated the breakpoint (BP) at 0.288 ± 0.088 % (95 % CI: 0.1155–0.4605 %) AP (Fig. 1b), while the one-slope quadratic ascending model provided a similar requirement estimate of 0.316 ± 0.166 % (Fig. 1c). Regarding FCR, the one-slope linear descending model estimated the BP at 0.282 ± 0.020 % (Fig. 1d), whereas the one-slope quadratic descending model indicated a requirement of 0.331 ± 0.036 % (95 % CI: 0.2604–0.4016 %) AP (Fig. 1e). Additionally, the two-slope linear descending–ascending model predicted the requirement for FCR at 0.290 ± 0.027 % (Fig. 1f). Based on lowest *S*_y.x_ and highest R^2^, the best estimation for weight gain and feed conversion ratio were 0.288 and 0.331 % AP, respectively.

The results demonstrate a direct and significant relationship between dietary AP and the growth performance of quail chicks from 7 to 14 days of age. The linear improvement in weight gain with increasing AP up to the breakpoint of 0.288 % is physiologically logical. Phosphorus is a fundamental component of hydroxyapatite, the primary mineral in bone, and is essential for the rapid skeletal development occurring during this life stage. Furthermore, its role in energy metabolism as a key component of adenosine triphosphate (ATP) is critical to fuel protein synthesis and tissue accretion. Therefore, as the dietary AP level increased from deficient to sufficient, the quails were better equipped with the structural and energetic resources needed for maximal growth, which explains the observed response. The estimated requirements for AP based on G and FCR ranged approximately between 0.288 % and 0.331 %. Specifically, the best fit for G was 0.288 % AP, while FCR was optimized at about 0.331 % AP. These results align with previous findings in Japanese quail and other poultry species where AP needs for maximal growth performance typically cluster within a narrow percentage range around 0.3 % to 0.4 % in starter diets. For example, Mariz et al. (2017) reported phosphorus recommendations for young quails close to these values, underscoring the importance of maintaining adequate phosphorus levels during critical early growth phases.

Similarly, the improvement in FCR, which was optimized at a slightly higher level of 0.331 % AP, can be attributed to the central role of phosphorus in metabolic efficiency. An optimal FCR requires not just tissue growth, but also efficient nutrient assimilation and energy transfer. Adequate phosphorus ensures that metabolic pathways function at peak efficiency, allowing the bird to convert feed into body mass more effectively. The higher requirement for FCR compared to weight gain suggests that while a certain level of phosphorus is enough to support tissue deposition, a slightly higher level is needed to fully optimize the underlying metabolic processes that govern feed efficiency. This phenomenon is consistent with research showing that phosphorus is integral to the function of numerous enzymes and coenzymes essential for nutrient utilization (Mello et al., 2012; Saraiva et al., 2009). Studies by Beiki et al. (2013) and Ribeiro et al. (2016) also concluded that adequate phosphorus levels enhance overall nutrient utilization, leading to improved feed efficiency in poultry. The use of multiple regression models, particularly the agreement between one-slope and two-slope models, strengthens the confidence in this identified requirement range.

It is important to acknowledge the limitations of the current study. First, the experiment was conducted over a short duration (7 to 14 days of age), and these findings may not be directly applicable to the entire starter period or subsequent growth phases. Second, our evaluation was focused on growth performance metrics (weight gain and FCR). We did not measure critical skeletal health indicators such as bone ash content or bone breaking strength, which could provide a more comprehensive understanding of the phosphorus requirement for both growth and skeletal integrity. Finally, the determined requirement is specific to the basal diet composition used in this study, including its specific calcium and phytate-phosphorus content. Variations in these dietary components could alter the optimal available phosphorus level.

In conclusion, the findings confirm that dietary AP is a pivotal factor for maximizing growth and feed utilization efficiency in growing quails during the second week of life. The optimal range for AP between 0.288 % (for weight gain) and 0.331 % (for FCR) dietary inclusion corresponds well with published literature and aligns with established mineral nutrition principles in poultry. Appropriate segregation of phosphorus requirements during phased feeding programs could optimize resource use, improve quail performance, and reduce environmental phosphorus loading.

## CRediT authorship contribution statement

**Mehran Mehri:** Writing – review & editing, Writing – original draft, Supervision, Formal analysis, Conceptualization. **Masoumeh Bameri:** Writing – review & editing, Resources, Investigation. **Mahmoud Ghazaghi:** Writing – review & editing, Validation, Funding acquisition. **Morteza Asghari-Moghadam:** Writing – review & editing, Funding acquisition, Data curation. **Hamid-Reza Behboodi:** Writing – review & editing, Visualization, Software, Resources. **Mohammad Rokouei:** Writing – review & editing, Methodology, Formal analysis, Conceptualization.

## Disclosures

The authors declare that they have no known competing financial interests or personal relationships that could have appeared to influence the work reported in this paper.
